# A Case Report on Endometriosis: “A Tip of Iceberg” Disease Identified and Managed Better With Imaging

**DOI:** 10.7759/cureus.40696

**Published:** 2023-06-20

**Authors:** Mukta Agarwal, Shivangni Sinha, Smita Singh, Sudwita Sinha, Ishita Roy

**Affiliations:** 1 Obstetrics and Gynecology, AII India Institute of Medical Sciences, Patna, Patna, IND

**Keywords:** pre-operative planning, image guided surgery, ureteric injury and repair, inflammation and infertility, endometriosis and chronic pelvic pain

## Abstract

Endometriosis has been more common in recent decades as a result of improved diagnosis supported by enhanced clinical concepts and greater imaging tools. A multidisciplinary care strategy that extends beyond the cellular level is required for this complicated pathophysiological condition to enable patients to live disease-free lives. This case study features a young woman who was diagnosed with endometriosis and was anxious about future fertility. The condition eventually led to a series of events that defined the intricacy of the sickness involved and the accompanying complications that were better detected, and so, therapy was comparatively simplified by imaging, which helped manage the ailment and its issues. The related complications are well established; however, with the impending use of 3D imaging technology, the disease, its extent, and associated complications can be managed in a well-planned manner.

## Introduction

Endometriosis governs a complex pattern of heredity that affects 30-60% of patients [1]. The condition is characterized by benign proliferative disorder with a propensity for invasion into surrounding tissues. The symptoms are progressive, ranging from no complaints to dysmenorrhea and persistent pelvic discomfort (15.4% to 71.4%), menstrual disorder, dyspareunia, dyschezia, and infertility (9% to 68.0%) [2]. This is a hormone-dependent disorder in which symptoms may not always correspond to the extent of lesions, complicating the therapy proportion.

Imaging techniques with different attenuations provide more in-depth knowledge of anatomical integrity and in finding atypical lesions, which frequently pose diagnostic issues. The cinematic rendering (CR) and volume rendering (VR) 3D image processing techniques of computed tomography (CT) image data have demonstrated promising applicability in researching complicated anatomical structures, and pre-operative and post-operative imaging in malignancy. Their routine use in the therapy of complex benign cases can produce the best results [3]. This purpose has been demonstrated by this case report. We discuss the clinical courses of a young patient diagnosed with grade 4 endometriosis and its aftermath. The case was efficiently treated with pre-operative imaging guidance in surgery planning and execution. Informed written consent for publication was obtained.

## Case presentation

A 30-year-old nulliparous woman presented to our outpatient department, anxious for an issue, complaining of acute dysmenorrhea that was progressing and coupled with dyspareunia. She had a history of visiting various hospitals in an attempt to treat her symptoms and conceive. The patient was in dismay and disheartened by prolonged medical treatment in the past.

Her overall physical and systemic examinations were normal, with a per vaginum examination indicating a bulky uterus (14*8*6 cm) with associated uterine tenderness, bilateral adnexal mass (6*4cm and 6*6cm, respectively), and Pouch of Douglas (POD) fullness with deep motion tenderness. Her preliminary tests revealed that she had a low ovarian reserve (AMH: 0.05ng/dL). An abdominal magnetic resonance imaging (MRI) revealed endometriosis with a diffusely enlarged adenomyotic uterus (16*8*6cm) (focal asymmetrical thickening of the junctional zone with multiple ill-defined areas of low signal intensity), and bilateral endometrioma (hyperintensity on T1W1 and low signal intensity on T2W1) corresponding to per vaginum examination (T2W1 imaging shows the fibrotic plaque on the serosal uterine surface with the tethered appearance of the rectum to the uterus suggesting obliterated POD). The fertility achievement with the IVF option and future disease prognosis was discussed.

The patient and her spouse were counseled, and consent was obtained for salpingectomy if necessary. She underwent laparoscopic adhesiolysis with adeno-myomectomy and bilateral cystectomy with her agreement. Intra-operative findings revealed dense adhesions involving the uterine surface, fallopian tubes, ovary, and pelvic surface. The anatomical integrity of the fallopian tube and ovaries was found to be altered. Bilateral tubes were tortuous and dilated, with a fimbrial end that was adherent to an adjacent structure. The tubal patency test was negative following which bilateral salpingectomy was done after re-informing detailed intra-operative findings to her husband. The postoperative phase was uncomplicated, and the patient was discharged on POD3.

**Figure 1:**
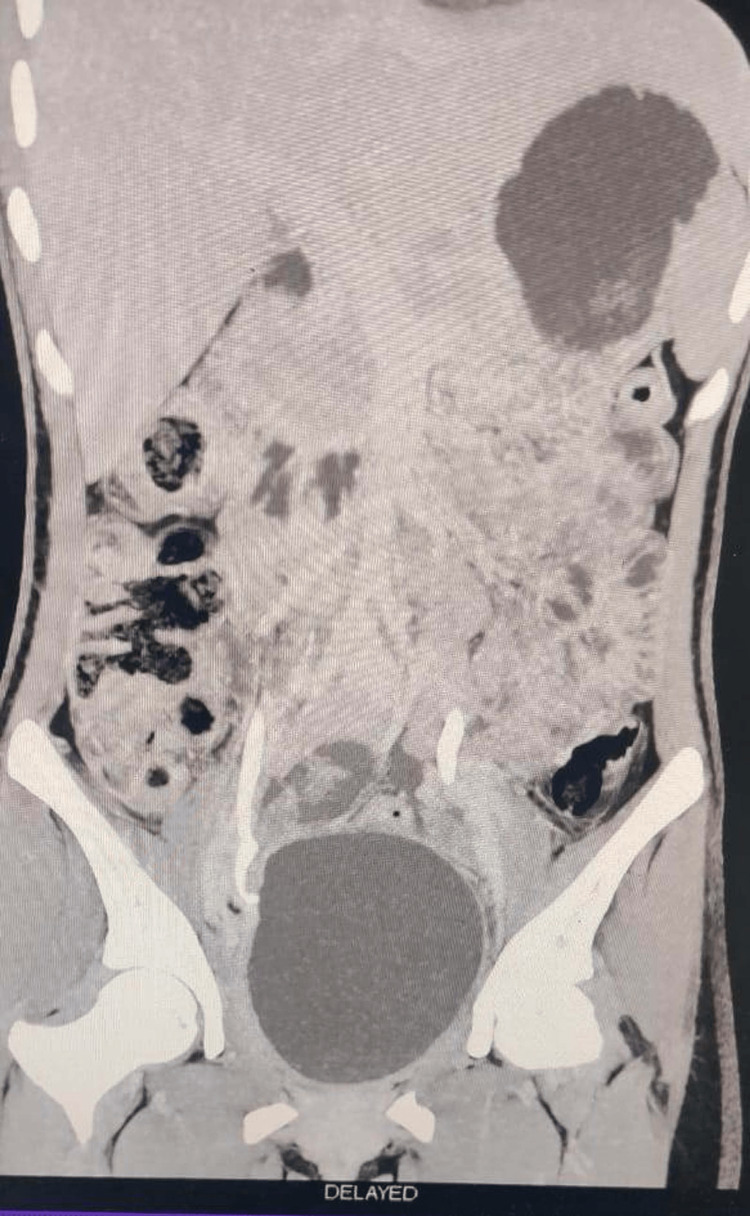
Coronal CECT image shows intra-peritoneal collection in the right lower quadrant with no air foci within with a distal ureter injury communicating with a fluid pocket extending within the recto-uterine pouch. Self-ownership is declared

**Figure 2:**
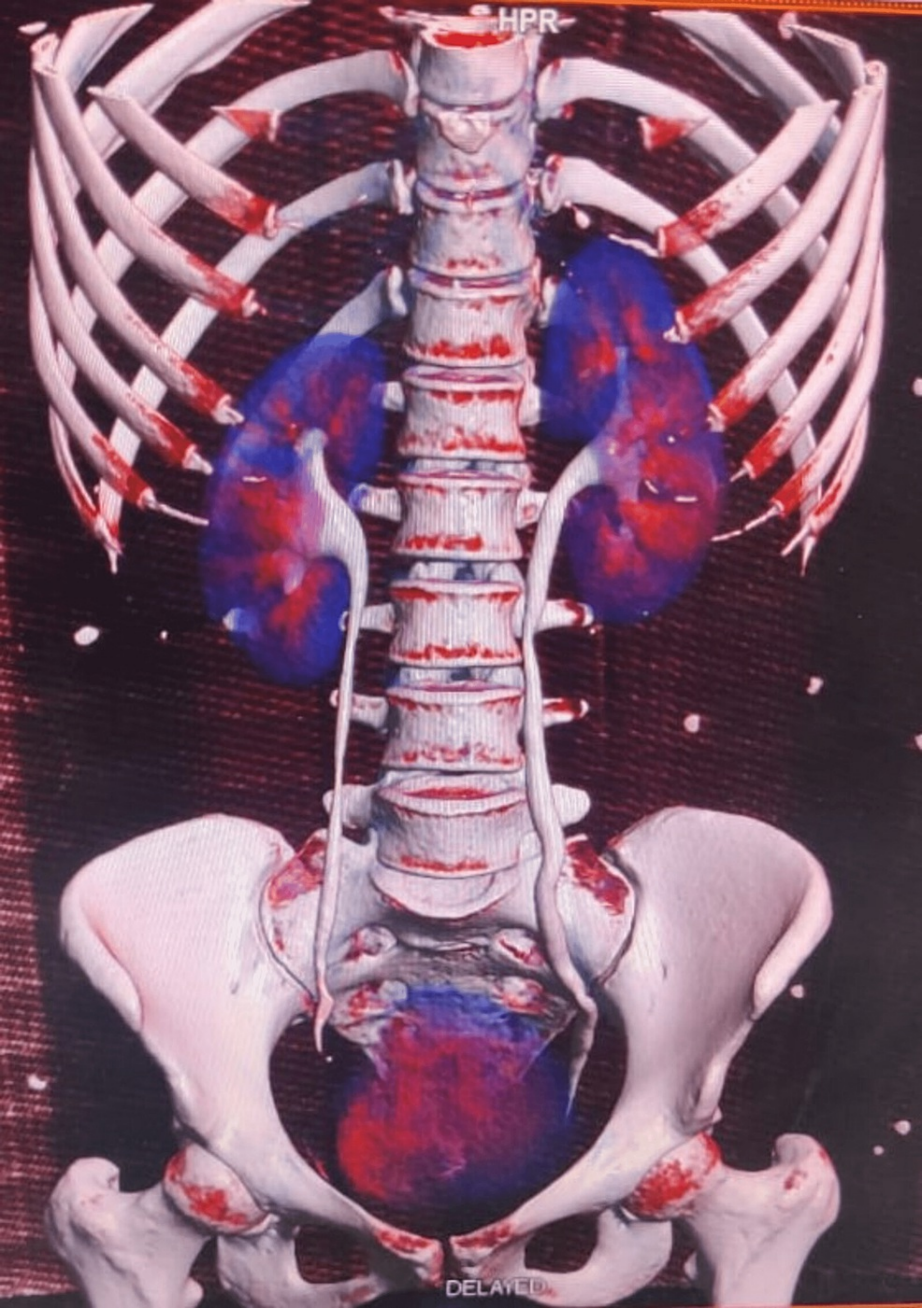
Coronal volume rendering images of the excretory phase show abrupt narrowing of the right distal ureter with mild proximal hydroureteronephrosis. Contrast-filled large walled-off urinoma in the pelvis. Self-ownership is declared

She presented to our emergency after 20 days after her discharge with complaints of high-grade fever, abdominal pain, and distention for seven days, not relieved of medication given by some local hospital. The patient was admitted. Immediate ultrasound was done that revealed around 600 mL collection in the right iliac fossa which was drained. The pus culture came sterile. Her condition remained the same with continuous high-grade fever with no better response to antibiotics following which she underwent CT Urography that showed a large walled-off intraperitoneal collection measuring 640cc in the right lower quadrant with no air foci within. A small rent in the distal right ureter at the level of S3 with a small fluid pocket extending within the recto-uterine pouch, terminal ureter not opacified likely sequel of right distal ureteric injury. A rent in the distal ureter at the level of S3 with a small fluid pocket extending within the recto-uterine pouch, terminal ureter not opacified, could be a possible consequence of right distal ureteric injury (Figures 1, 2). Also, the CT-guided drainage of fluid came positive for creatinine with 33.19 mg/dL. 

**Figure 3:**
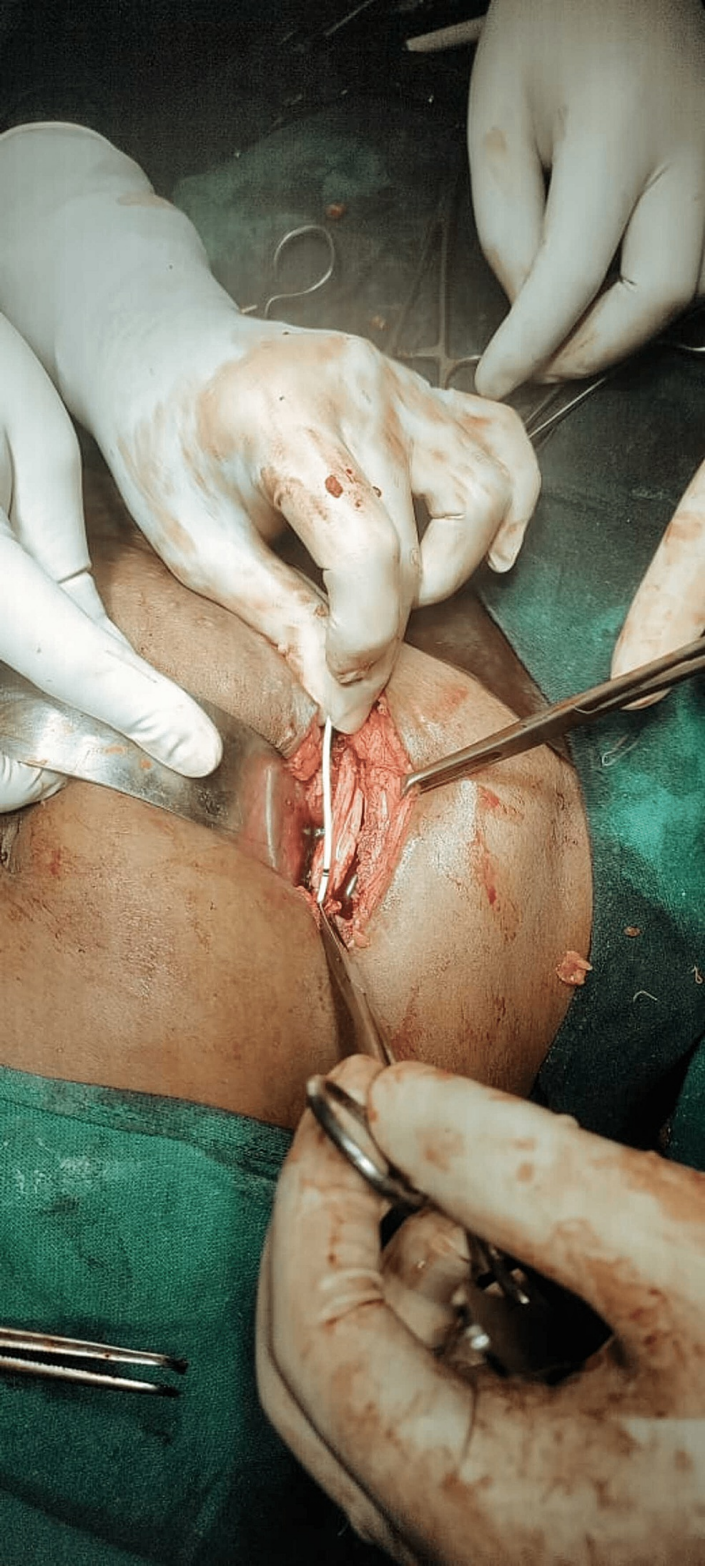
Intra-operative image shows right-sided DJ stent application during repair of ureteric injury. The urinoma was drained followed by re-anastomosis after adequate dissection. Self-ownership is declared

The patient was taken in for an emergency exploratory laparotomy with urinoma drainage and right ureteric re-implantation with psoas hitch of the urinary bladder. Intraoperatively, a lower ureteric defect 4cm from the vesico-ureteric junction was discovered, along with urinoma development in the transverse plane. The damaged ureter and its surroundings were found to be necrotic. Following proper dissection, a re-anastomosis was performed using a DJ Stent 6F 26cm that was left in place. The intra-operative findings were consistent with the pre-operative findings, assisting in the pre-planning of the surgery (Figure 3).

Following surgery, the patient showed recovery. However, starting on the fourth day, the patient developed a low-grade fever and pus discharge at the stitch site. The wound gap culture sensitivity study revealed that MRSA (methicillin-resistant Staphylococcus aureus) was sensitive to colistin. Antibiotics were prescribed, along with twice-daily sterile dressing changes at the wound site. After 10 days of dressing, good granulation tissue was observed, and the repeat wound swab culture became sterile. She was taken up for secondary suturing. On the 12th day after secondary re-suturing, the stitches were removed, and the patient was discharged. At the time of discharge, a high protein diet, cleanliness, and an abdominal binder were recommended with further follow-up in an IVF center for conception.

## Discussion

Endometriosis can affect fertility in a variety of ways, including distorted pelvic anatomy, adhesions, scarred fallopian tubes, inflammation of pelvic structures, altered immunity, changes in the endocrine environment of the endometrium and ovum, impaired pregnancy implantation, and altered egg quality. A complicated web of humoral, cellular immunological, and inflammatory implants and factors like interleukins, activated macrophages, tumor necrotic factors, and prostaglandins present in the peritoneal fluid impair the functioning of the oocyte, ovum pickup, motility, sperm quality, and endometrium affecting fertility [4]. It has been linked to an increased incidence of depression and anxiety disorders. Women who experience pelvic discomfort report significant levels of worry and sadness, a loss of workability, limitations in social activities, and a poor quality of life that places the onus on the treating physician to assist them in overcoming their impairment. Endometriosis may be caused or exacerbated by stress [5]. A Swedish study of 400 endometriosis patients discovered that “32% of the women reported absence from work, while 36% reported reduced time at work due to endometriosis” [6]. Another cross-sectional study of Puerto Rican women discovered that “endometriosis-related and concomitant symptoms impeded all aspects of women's daily lives, including physical constraints that affected doing household chores and professional employment." The majority of women (85%) reported a drop in work quality; 20% reported being unable to work due to discomfort, while more than two-thirds of the sample continued to work despite their pain [7].

Chronic debilitating pain lets patients choose surgical management (fertility-sparing and non-sparing treatment). Also, it is more effective than medication in treating endometriosis-related infertility where laparoscopic surgery is the preferred approach over open [8]. There are cohort studies that show surgery is beneficial in relieving pain [9]. Pelvic fibrosis in grade 4 endometriosis is one of the primary causes of ureteral damage during adhesiolysis laparoscopic surgery. They induce persistent inflammation and adhesions, altering pelvic architecture, and can involve the ureter (extrinsically or intrinsically), shifting the ureter from its anatomical location, making assessment difficult, and causing injury. Its prevalence rises from 0.2% in gynecological surgery to up to 1% in endometriosis [1,10]. The injury is normally detected three to five days after the procedure, but if ignored, it can lead to irreversible renal failure. The risk of ureteric injury can be reduced by using a discrete energy source across the path of the ureter, which is topographically related to the common iliac vessel, round ligament, and obliterated medial umbilical ligament. To avoid injury, adhere to the thumb rule of staying medial to the obliterated umbilical ligament [11].

Chronic inflammation initiates some signaling pathways leading to necrosis and reduced immunity in endometriosis causing increased risk of infections (Genito-urinary), chronic endometritis, and surgical site infections. Infection with Gardnerella, Streptococcus, Enterococci and Escherichia coli, mollicutes, and shigella has been found associated [12,13]. Evidence suggests auto-immunity and endometriosis share a common pathway that shows strong potential for research to formulate immunostimulatory drugs that can prove effective [14]. Research works are being done to identify non-invasive imaging markers that would help early diagnosis and triage cases of endometriosis that require surgical management, and also cases that can be provided non-surgical management that would complement their pathophysiological diagnosis along with imaging techniques and laparoscopic surgeries [15].

Imaging technologies have improved understanding of the extent of problems by giving a precise vision of complicated anatomy, vascular structures, and information on tissue levels such as fibrosis and edema [16]. The 3D image processing techniques of computed tomography (CT) image data have shown promise in investigating challenging anatomical structures, and pre-operative and post-operative imaging in benign and malignant conditions both [17]. Its routine usage would provide a better grasp of the disease's complexity and an efficient pre-planned operational approach to disease management and associated complications.

The case demonstrates the necessity of a multidisciplinary approach in the diagnosis and management of severe chronic pathophysiological disorders, which aids in the provision of effective quality care. The use of image-guided techniques aided not only in diagnosis but also in the planning of post-operative problems. This would assist doctors in selecting the approach that best meets the surgical goal while also maximizing patient safety. With an increased likelihood of recurrence following surgery, the length of time since surgery, and the stage of disease detected at the time of surgery, more effort is required to be able to give effective and longer-lasting disease-free existence with enhanced quality of life.

## Conclusions

Endometriosis is a mysterious disorder that can bring both physical and emotional agony to a patient disguising its presentation and dynamics. There is a longstanding connection between endometriosis and infertility; but, as indicated above, it appears to be multidimensional, with mechanical, molecular, genetic, and external factors all playing a role. The condition has no cure and is difficult to treat, with a high rate of recurrence even after surgery. The most appropriate therapy for endometriosis-related infertility is an individualized choice that should be decided on a patient-by-patient basis. To cope with such a complicated pathogenetic illness, a multidisciplinary approach has become essential to achieve better results. In addition to anatomical knowledge, the routine use of a visual guide provides a better understanding of the illness process and allows patients to have safer, more efficient, and well-planned surgical options, reducing associated morbidity.

It is a concept that requires a lot of research work, finally to be able to help patients have disease-free life with minimum sacrifices. Due to associated surgical difficulties and current information gaps, there is a need for non-invasive diagnostic approaches as well as medical therapies that do not inhibit pregnancy while also providing effective essential treatment.
